# Effect of Staining Solutions on Color Stability of Silorane & Methacrylate Restorative Material

**Published:** 2015-03

**Authors:** Prashanthi S. Madhyastha, Dilip G. Naik, Ravindra Kotian, N. Srikant, Kumar M. R. Bhat

**Affiliations:** 1 Department of Dental Materials, Manipal College of Dental Sciences, Mangalore, India;; 2 Department of Periodontics, Manipal College of Dental Sciences, Mangalore, India;; 3 Department of Oral Pathology, Manipal College of Dental Sciences, Mangalore, India;; 4 Department of Anatomy, Kasturba Medical College, Manipal University, Manipal, India

**Keywords:** Silorane based composites, Methacrylate based composites, Color stability, Staining solutions, Spectrophotometer

## Abstract

Color stability throughout the functional lifetime of restorations is important for the durability of treatment and of cosmetic importance. The purpose of this study was to evaluate the discoloration properties of a silorane-based (Filtek P90) and methacrylate-based (Z100) composites upon exposure to different staining solutions that are used on day to day basis (turmeric, tea, coffee, cocoa, lime, yoghurt and distilled water) for different immersion periods (1, 7, 14 and 28 days). The colors of all specimens before and after storage in the solutions were measured by a reflectance spectrophotometer based on CIE Lab system and the color differences were calculated. Data were statistically analyzed by repeated measures of ANOVA and sidak post hoc test (for immersion period);‘t’ test (for each material) and one way ANOVA (for staining agents). All the staining agents showed significant difference in staining over time in both the materials. However, Z100 showed higher quantum of discoloration at all time periods at each staining agents (*p*<0.005). In conclusion, the silorane-based resin (Filtek P90) composites exhibited better color stability (less change in Δ*E*) after exposure to the staining solutions. Among the staining agents cocoa was found to be least staining followed by lime, yoghurt, coffee, tea whereas turmeric discolored the composites to the maximum. Highest discoloration was seen at day 28 in all staining agents. Cocoa and lime discolored to maximum at early stages but remained stable thereafter whereas tea, coffee and turmeric progressively discolored the composite over time.

## INTRODUCTION

Tooth colored composite resins with the methacrylate chemistry have been commonly and successfully used in restorative dentistry. They mainly comprise of monomeric resin matrix, silinated inorganic filler, polymerization initiator system, inhibitors for storage stability and pigmentation for shading (1, 2). The inevitable problems of polymerization shrinkage (2-3% by volume) and its clinical outcomes like micro leakage, decrease in marginal integrity, microcracks, debonding, postoperative sensitivity could be overcome by changing the chemistry of setting and using a composite based on silorane chemistry. This lead to the development of new innovation in the field of dental composites. A silorane based composite (Filtek P90), comprising of ring-opening monomer, with siloxane and oxirane structural moieties, have been introduced claiming low polymerization shrinkage (>1%) and also with improving biocompatibility, wear resistance, optical properties. This low shrinkage because of cationic ring opening polymerization process of cyclic epoxides is desirable in the development of a new dental composite filling material superior to the current resin compounds based on acrylates (1).

The success or failure of any esthetic restorative material mainly depends on color match and the color stability of material in long term use. Color stability, the property of material to retain its original color over a period of time, in a specified environment, is an important property of many materials used in dentistry (3). However composites resins suffer in-service discoloration (staining) with time leading to patient dissatisfaction, additional time and money for replacement of restorations (4). Several studies in literature have implicated dietary compounds as major etiological factor in staining of composite restorations that remain in prolonged contact with the dental surfaces (4-10). The effects of such dietary compounds on methacrylate based resins have been investigated before, but have not been thoroughly studied for silorane based composite. Effect of the storage period on these dietary compounds (staining agents) has not been reported earlier. Therefore the objectives of this study were to evaluate the effect of staining solutions and immersion time on color stability of silorane restorative material (Filtek P90) in comparison with its methacrylate counterpart (Z100).

## MATERIALS AND METHODS

In this study, six commonly consumed beverages were used as staining agents and distilled water as control, to evaluate the effects of color stability of silorane restorative material (Filtek P90) in comparison with its methacrylate counterpart (Z100).

Table 1 and Table 2 lists the details of the resins and staining agents used in the present study. It is known that obtaining conditions of oral environment *in vitro* is not easy. To simulate *in vivo* conditions in this study, six different staining solutions and distilled water (control) were used at a constant temperature of 37 ± 1°C. The quantities of staining ingredients to make the test solutions were prepared closer to the amount consumed per person at a time. This study is an indicator of cumulative effect of repeated short immersions of composite resins used for restorations, during prolonged service (11, 12).

**Table 1:** Materials used for the study

Name	Type	Manufacturer	Shade	Organic matrix	Inorganic fillers	Filler content [wt.%]

**Z100**	Methacrylate based universal composite	3M/ESPE, St. Paul, MN, USA	**A2**	Bis-GMA and TEGDMA	Zirconium, silica	66
**Filtek P90**	Silorane based microhybrid composite	3M/ESPE, St. Paul, MN, USA	**A2**	3,4-Epoxycyclohexylethylcyclopolymethylsiloxane, bis-3,4-poxycyclohexylethylphenylmethylsilane	Silanized quartz, yttrium fluoride	76

**Table 2:** Staining agents used in the study

Staining Agents	Code	Manufacturer	Proportion

**Turmeric**	T	MTR Food Ltd, Bangalore, India	1g/ltr boiling water, simmer for 5 min & filtered
**Coffee**	C	Nescafe Sunrise Premium, Nestle India Ltd, New Delhi, India	30g/ltr boiling water, simmer for 5 min & filtered
**Tea**	E	Brooke Bond Red Label Hindustan Unilever Ltd Mumbai, India	30g/ltr boiling water, simmer for 5 min & filtered
**Cocoa**	O	Cadbury Cocoa, Cadbury India Ltd, Mumbai, India.	30g/ltr boiling water, simmer for 5 min & filtered
**Lime**	L	Home made	100ml/ltr of distilled water
**Yoghurt**	Y	Home made	

### Preparation of test solutions

30 g of tea, coffee and cocoa and one gram of turmeric were separately taken in one liter of distilled water and solution was prepared by boiling, followed by simmering for five min and filtered through a filter paper. In case of lime, 100 ml of lime juice concentrate was dissolved in one liter of distilled water (11, 12).

### Preparation of test specimens

Composite specimens were prepared using Brass molds (10 mm diameter and 2mm thickness) and cured for the duration recommended by manufactures. A total of 150 samples (70 samples for each group of P90 & Z100 resin, [n=10]) were fabricated which were to be stained for 1, 7, 14 and 28 days’ time interval. All the specimens were coded to avoid manual bias while evaluating through spectrophotometer. The samples were polished with different grades of Silicon Carbide paper (80, 100, and 120) and buff polished with pumice slurry.

### Method

The resin samples were stored in distilled water for 24 hour before immersing into the test solutions (13). After the baseline measurement, the samples were immersed in staining agents: Turmeric, Yoghurt, Coffee, Tea, Cocoa and Lime for 8 hour of immersion period daily (simulating overnight soaking period) for 1 day, 7 days, 14 days and 28 days at 37°C. The solutions were stirred twice a day to reduce precipitation. The solutions were refreshed each day. The samples were then rinsed in distilled water for 8-10 dips and blot dried with a tissue paper. They were stored in a desiccator for the remaining period until they were placed in a viewing port for color measurement. The control samples were stored in distilled water at 37°C, the water being changed every day.

### Measurement of color

Color changes (ΔE) were measured objectively by a reflectance spectrophotometer (Spectrolino, Gretag-Macbeth AC, Germany), to potentially eliminate the subjective errors of color assessment. To measure chromatic differences Standard Commission International de L’ Eclairage (CIELab) color system was used. It quantifies color in terms of three coordinate values L^*^, a^*^ and b^*^. L^*^ represents brightness or lightness (value), a^*^ and b^*^ serve as numeric, correlates both for hue and chroma on green- red axis and blue- yellow axis respectively. The magnitude of color difference perceived between two objects is calculated by formula ΔE = (ΔL^2^ + Δb^2^ + Δa^2^)^½^.

Before each measurement session, the instrument was calibrated according to manufacturer instruction by using the supplied white calibration standard. Color was then measured according to CIE Lab color scale. D50 standard illuminant from a tungsten lamp was used with a viewing angle of 2°. UV filter was positioned to 100% UV. Color measurements were made in three randomly selected areas for each specimen and average of three reading was recorded. The mean and standard deviation was calculated.

### Statistical analysis

Data were statistically analyzed by repeated measures of ANOVA and sidak post hoc test (for immersion period);‘t’ test (for each material) and one way ANOVA (for staining agents). The data was expressed as mean ± SD. p values < 0.05 was considered as significant.

## RESULTS

**Figure 1:**
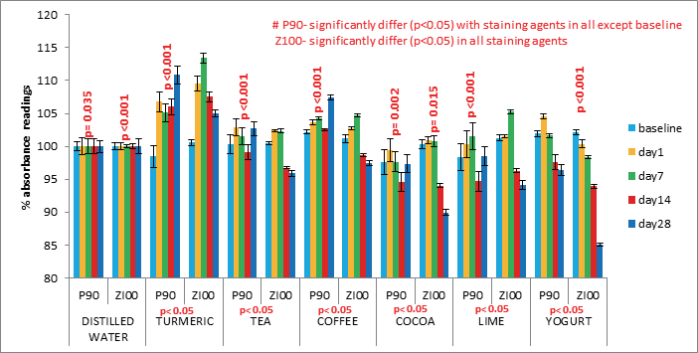
Percentage absorbance readings of P90 and Z100 in various staining solutions at different time intervals.

Composite P90 (Figure 1) showed increased discoloration from day 1 to day 28 in all fluids except cocoa, yogurt and lime. Composite Z100 had a trend of increased discoloration (*p*<0.05) from baseline to day 7 and then decreased to day 28. Large number of significant differences was seen in comparison with various staining agents. In both the materials the highest discoloration (*p*<0.05) was seen with turmeric and least with cocoa. Composite P90 showed similar trend in yoghurt, coffee, tea, lime in decreasing order. Also it was observed that coffee and turmeric shows consistent increase (*p*<0.05) from baseline to day 28. Z100 showed higher discoloration (*p*<0.05) in lime followed by coffee, tea, yogurt.

In conclusion, the silorane-based resin (FiltekP90) composites exhibited better color stability (less Δ*E*) after exposing to staining solutions. Among the staining agents cocoa was found to be least staining substance followed by lime, yoghurt, coffee, tea. The turmeric was found to be the most discoloring agent on both the composites. Highest discoloration was seen at day 28 in almost all the staining agents. Cocoa and lime discolored the composite to maximum at early stages but remained stable thereafter. However, tea, coffee and turmeric progressively caused discoloration of the composite over time.

## DISCUSSION

Color stability throughout the functional lifetime of restorations is important for the durability of treatment. Color alterations in dental composites are multifactorial and are associated with *intrinsic factors* like chemical changes in the material (like resin matrix- filler particle content) (6, 14) and at the matrix/particle interface (15) and *extrinsic factors* like adsorption or absorption of stains/exogenous sources, dietary, smoking habits (7, 10) and water sorption coefficient of resinous monomers (16-18). Consumption of certain beverages affects the esthetic and physical properties of resin composites, compromising on the quality of restorations. In the present study, when discoloration of two resin composites was compared, the overall maximum discoloration was seen in methacrylate based composite (Z100) when compared with silorane based composite (Filtek P90) and the results were statistically significant. It is considered that color alteration values of ΔE greater than or equal to 3.3 are visually perceptible and clinically unacceptable to 50% of the trained observers (19). In the present study, both P90 and Z100 showed perceivable color changes, but composite P90 showed significantly lower color alterations in comparison with the Z100 composites (*p*<0.05).

The possible explanation for the differences between silorane and methacrylate-based composites is that the methacrylate based composites have higher water sorption (20, 21), induces plasticization and expansion of the methacrylate polymer (22) and have hydrophilic monomer (TEGDMA which have been reported to stain more readily) (4, 20) compared to silorane resins. Siloranes are extremely hydrophobic, (because of the inaccessible siloxane groups to water or water- soluble species) (23), has lower hygroscopic expansion and higher dimensional stability in water (22). Also, the increased synergism between filler particles and resin matrix (siloxane component) may be responsible for the reduction in water sorption and solubility (24) This hydrophobic attribute of siloxane therefore favors color stability and minimizes the staining in silorane composites (4, 25). In addition to its hydrophobic properties, the silorane is stable and insoluble in simulated biological fluids containing citric acid, hydrochloric acid, heptanes, or enzymes such as hydrolase or esterase (23, 26) and artificial ageing (6). Further, it has been reported that resin composites with a lower amount of inorganic fillers showed more color changes because a greater resin matrix volume allows greater water sorption (27, 28). The lesser filler composition of Z100 corresponds to the greater color variation observed in the present study. Although there are conflicting results saying that not all the physical and mechanical properties of these new composites present satisfactory results as they say that color stability is also dependent on internal factors (7, 14, 29) and time (ageing) (7, 30).

Previous studies demonstrated that silorane was less susceptible to staining, compared to methacrylate- based composites, when immersed in red wine, coffee, soft-drinks, tea, and whisky (4, 10, 25). However the effect of these staining agents on properties of composite resins depends on length of immersion, the temperature, and pH of immersion solution (26, 31). Polymeric materials reportedly display a tendency to erode under acidic conditions (32-34). In general, food stuffs with low pH have greater erosive effect. Low pH affected the surface integrity of polymers. This was because under acidic conditions, the polymer surface was appreciably softened by loss of structural ions. Yoghurt and lime exhibited a similar behavior. The acidic ingredients in yoghurt and lime for eg. lactic acid and citric acid might have caused surface dissolution of polymeric surface leading to much paler appearance. Hence specimens after their specified immersion period when observed visually showed a lighter color match when compared to control.

In the present study, among all the staining agents used, Turmeric showed highest color changes in day 28 time interval (*p*<0.05). It is said that major constituents of turmeric (Curcuma) are curcuminoids, the yellow coloring principles that cause stain. Smaller molecular size of curcumin coupled with the water absorption characteristics of the tested materials has created a stronger staining effect as discussed by Ergun *et* *al.* 2005 (5). When considering the discoloration in coffee and tea, the specimens immersed in tea showed more discoloration. This is in agreement with the other studies in literature (35, 36). Um and Ruyter *et* *al.* 1991 (35) reported that the tea produced a yellow- brown stain while coffee stain was yellowish. The discoloration in tea was mainly due to surface adsorption of polar colorants at the surface. However other studies (20, 37-40) found that coffee was more chromogenic than tea. The discoloration by coffee is due to both surface absorption and adsorption of colorants. Fine coffee particles deposits into pits that may have formed due to polymerization shrinkage of resin during curing. The less polar colorants and water soluble polyphenols in coffee for eg. Tannin, caffeine, caffeinic acid might have penetrated deep into the material, possibly because such colorants could be more compatible with polymer matrices. When cocoa was considered there was less change in discoloration values. This result is attributed to the removal of accumulated layers. As the cocoa layers on specimen reached a certain thickness they tend to break away from the surface of samples and return to the solutions. This was the reason for less staining characteristics in cocoa. Therefore, colour changes due to extrinsic factors may be directly related to the organic matrix specifically present in the particular composite, and staining could thus be reduced in more hydrophobic materials.

Time was found to be a critical factor for the color stability of tooth colored restorative materials (41). In the present study, results showed that as the immersion time increased, the color changes became more intense.

The results of this study can give an insight into how different resin composites may behave when exposed to different beverages, thus affecting the clinician’s choice of the material and the patients control of dietary habits. Also the results showed that the effect of interaction of different composites, various beverages and time, depended on a multitude of factors. However, to investigate the color stability performance of composites in a clinical setting, these results should be supported by planned *in vivo* studies.

## CONCLUSION

From the present study we can conclude that, color stability is of great importance to patients and clinicians when working in the esthetic zone. Patients should be aware of their dietary habits if their restorations needs to be worn for long period and therefore, may be advised to avoid or minimize consumption of these beverages during the service of the composites. The dentist should select and use materials with good color stability, for the excellent serviceability of these restorations.
